# Supplementary material for: Dynamic assembly of a large multidomain ribozyme visualized by cryo-electron microscopy
Source: Nat Commun. 2025 Nov 27;16:10195. doi: 10.1038/s41467-025-65502-8 (PMC12660903; doi:10.1038/s41467-025-65502-8)
Supplement: Supplementary file 1 — Supplementary Information [file 41467_2025_65502_MOESM1_ESM.pdf]

SUPPLEMENTARY INFORMATION FILE FOR:

Dynamic assembly of a large multidomain ribozyme visualized by cryo-electron microscopy

Shekhar Jadhav[1,7], Mauro Maiorca[2,3,4,#], Jacopo Manigrasso[5,6,#], Spandan Saha[7], Auriane Rakitch[7], Stefano Muscat[5], Thomas Mulvaney[2,3,4], Marco De Vivo[5,*], Maya Topf[2,3,4,*], Marco Marcia[1,7,8,9,*]

[1]European Molecular Biology Laboratory (EMBL) Grenoble, 71 Avenue des Martyrs, Grenoble 38042, France

[2]Centre for Structural Systems Biology, Notkestraße 85, Building 15, D-22607, Hamburg, Germany

[3]Leibniz Institute of Virology, Martinistraße 52, 20251, Hamburg, Germany

[4]University Medical Center Hamburg-Eppendorf, Martinistraße 52, 20246, Hamburg, Germany

[5]Laboratory of Molecular Modelling & Drug Discovery, Istituto Italiano di Tecnologia, Via Morego 30, 16163, Genoa, Italy

[6]Current address: Medicinal Chemistry, Research and Early Development, Cardiovascular, Renal and Metabolism (CVRM), BioPharmaceuticals R&D, AstraZeneca, Gothenburg, Sweden

[7]Department of Cell and Molecular Biology, Uppsala University, Husargatan 3, 75123 Uppsala, Sweden;

[8]Istituto Italiano di Tecnologia, Via Morego 30, 16163, Genoa, Italy

[9]Science for Life Laboratory, Department of Cell and Molecular Biology, Uppsala University, Husargatan 3, 75123 Uppsala, Sweden

[#]Equally contributed

*Corresponding authors:

Dr. Marco Marcia, Phone: +46761162792, E-mail: marco.marcia@icm.uu.se

Dr. Marco De Vivo, Phone: +390102897404, E-mail: marco.devivo@iit.it

Dr. Maya Topf, Phone: +4940899887660, E-mail: maya.topf@cssb-hamburg.de

This Supplementary Information File includes:

- Supplementary Figures S1-S14
- Supplementary Tables S1-S6

SUPPLEMENTARY FIGURES

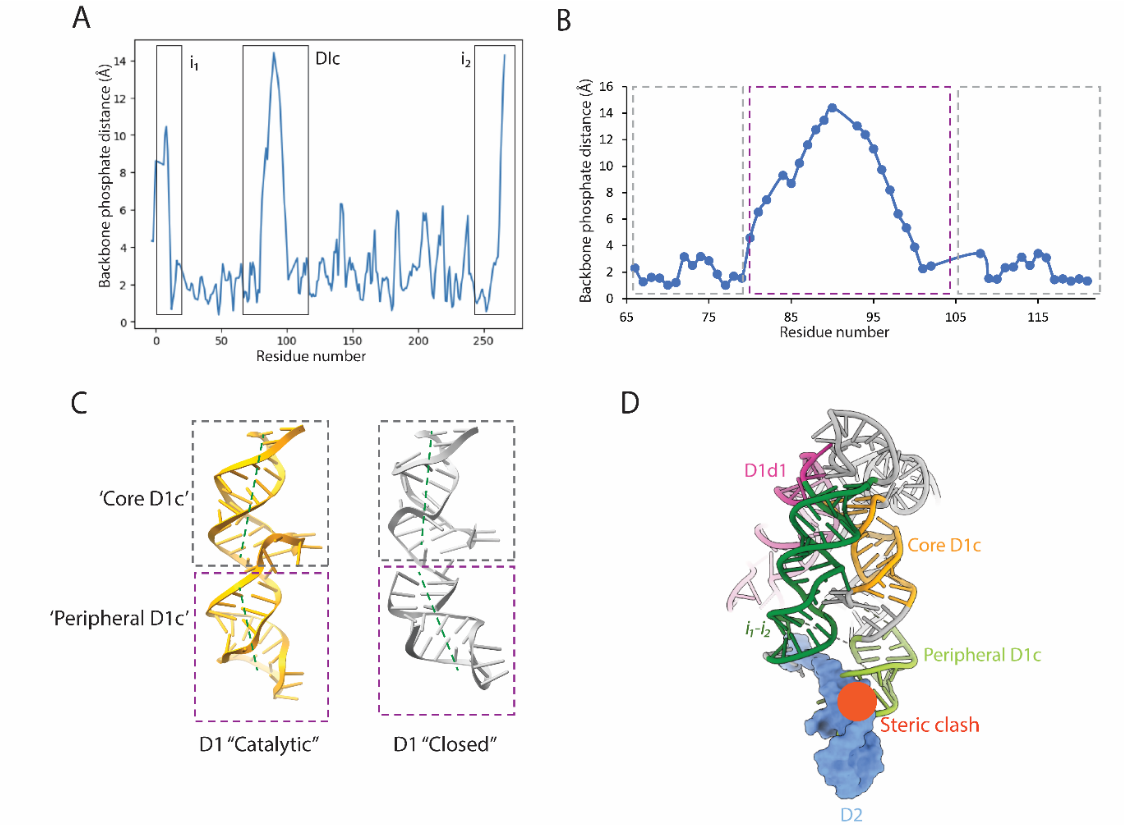

Supplementary Figure 1. Dynamics of helix D1c. (A) Pairwise phosphate atom distances between the "closed" and "catalytic" states of D1. The regions highlighted with black rectangles indicate helices D1c and i_1-i_2. Source data are provided as a Source Data file. **(B)** Zoomed in view of the pairwise phosphate atom distances of helix D1c. 'Core D1c' is depicted in grey, 'peripheral D1c' is depicted in magenta. **(C)** Helix D1c in the "catalytic" (orange) and "closed" (grey) states. The dashed boxes identify the 'core D1c' and 'peripheral D1c' motifs. The dashed lines represent the helical axes of these two motifs. **(D)** Steric clash between 'peripheral D1c' from the D1 crystal structure (PDB id 4Y1O) and D2 from the D1-5 crystal structure (PDB id 4FAQ). The region of the clash is indicated by the red dot.

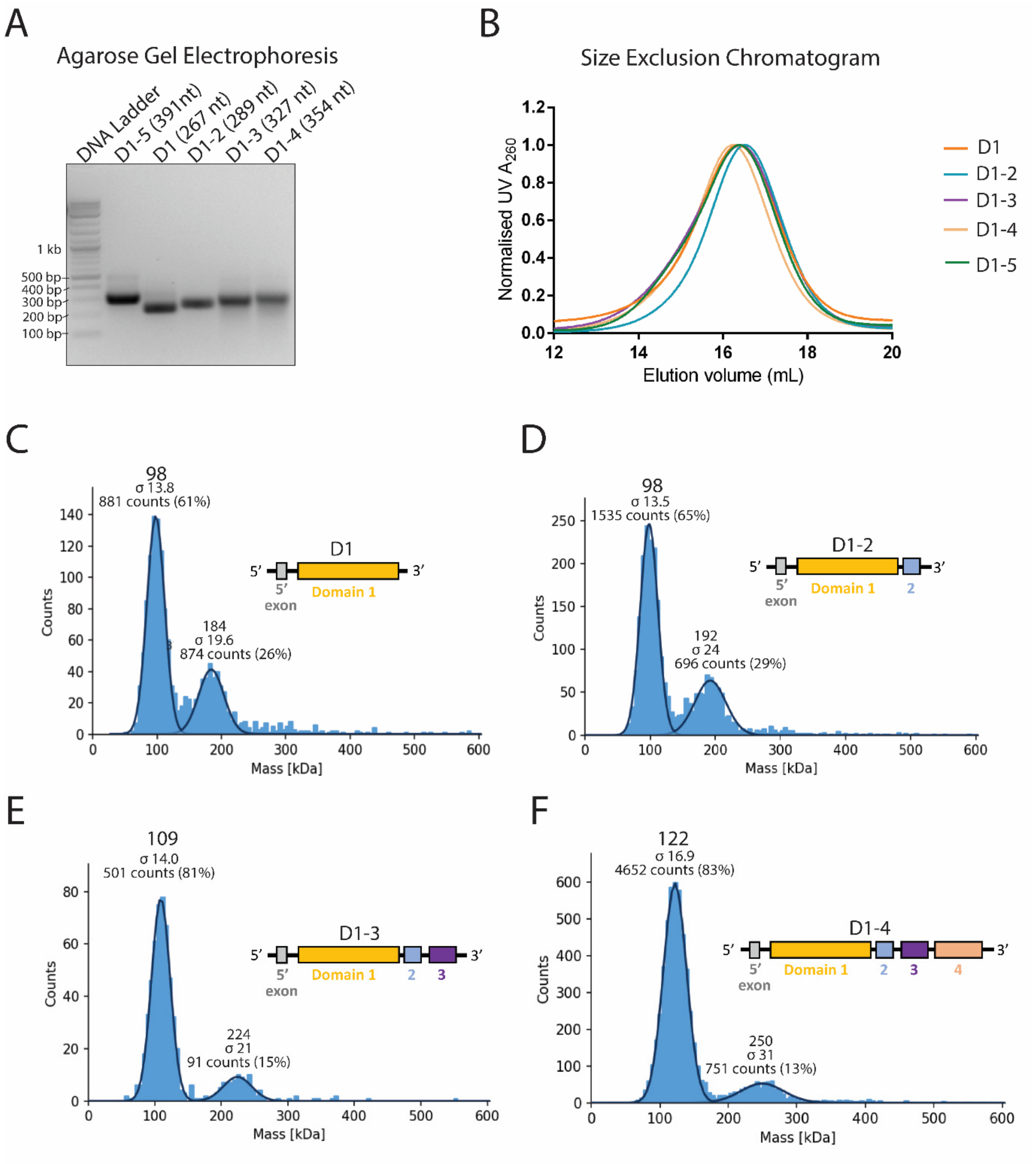

Supplementary Figure 2. RNA purification and biophysical characterization. (A) Native agarose gel electrophoresis of all purified *in vitro* transcribed RNAs used for structural characterization in this work. **(B)** SEC elution profiles of the same constructs. **(C-F)** Mass distribution acquired from MP for the same constructs. The mass distribution plot in panels C-F reveals also a minority of larger molecular weight forms of the RNAs. These forms are likely technical artefacts resulting from the high sensitivity of the MP instrument, and due to the binding of two molecules very close to each other on the measurement surface. Source data are provided as a Source Data file.

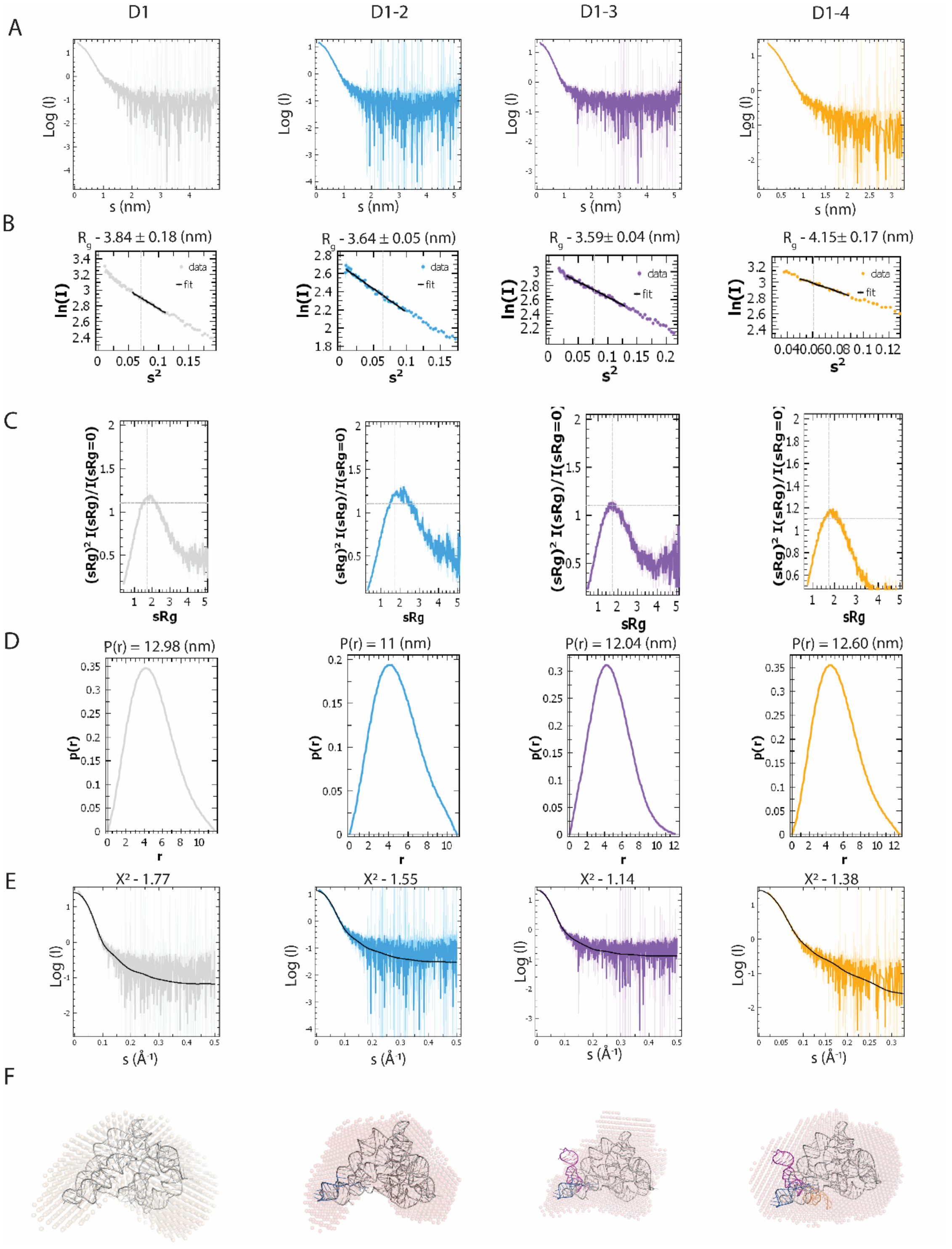

Supplementary Figure 3. SEC-SAXS analysis. (A) Scattering curves of the constructs indicated above the graphs. **(B)** Guinier plots. **(C)** Kratky plots. **(D)** Pairwise distance distribution plots. **(E)** Crysol plots. **(F)** Superposition of Dammif bead model derived from the SAXS dataset and corresponding coordinate models. The coordinate model of D1 is from PDB id 4Y1O. The coordinate models of all other constructs are derived from coordinates extracted from PDB id 4FAQ.

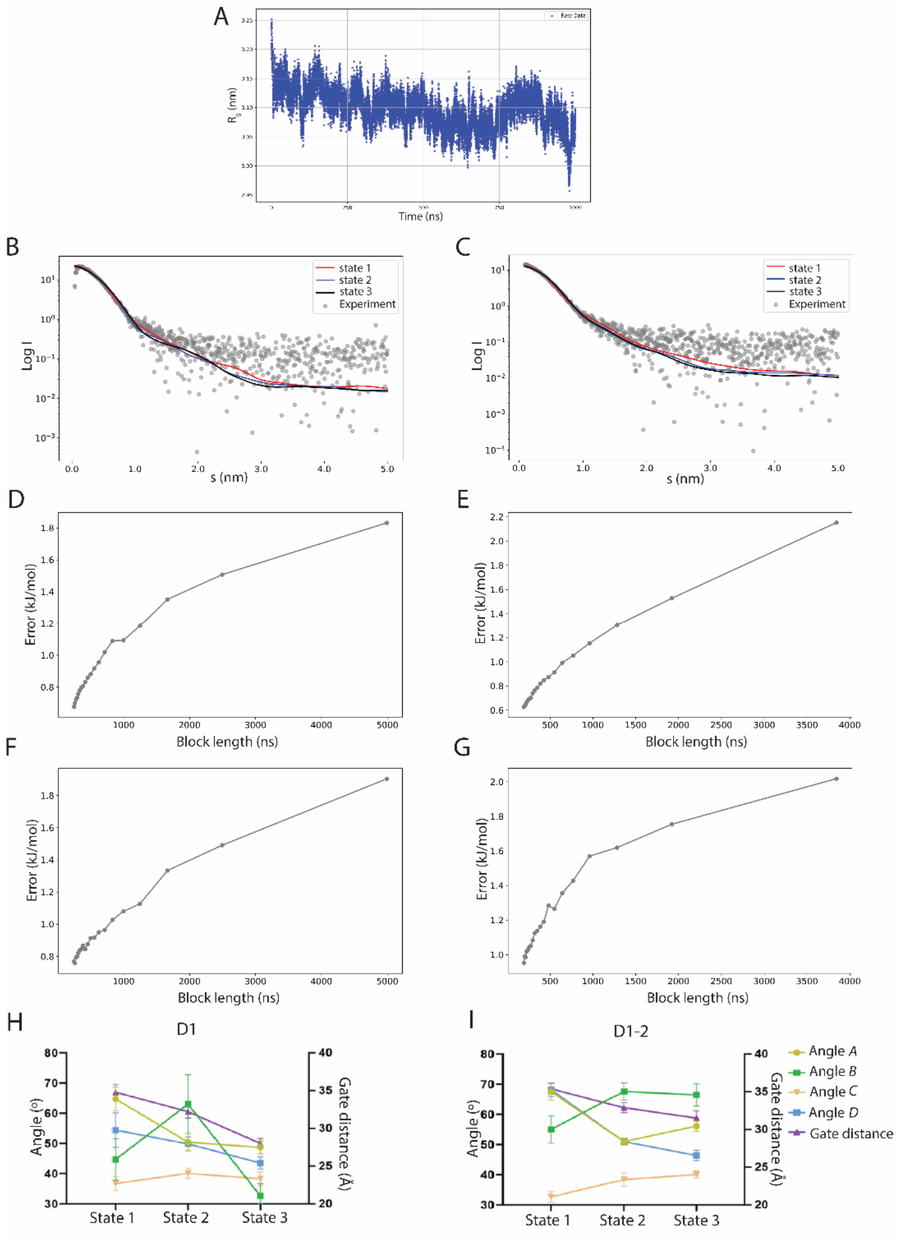

Supplementary Figure 4. SAXS-driven metainference metadynamics simulations and structural analysis. (A) Equilibrium MD simulations of isolated D1 in the presence of calcium ions. **(B)** Theoretical scattering curves derived from representative frames of the three free energy minima identified by the metainference metadynamics simulations (red, blue, and black, respectively) with the experimental SAXS curve (grey) for the isolated D1. For D1, the χ^2 of state 1, state 2, and state 3 are 1.09, 1.83, and 1.93, respectively. **(C)** Theoretical scattering curves derived from representative frames of the three free energy minima identified by the metainference metadynamics simulations (red, blue, and black, respectively) with the experimental SAXS curve (grey) for D1-2. For D1-2, the χ^2 of state 1, state 2, and state 3 are 1.23, 1.67, and 2.20, respectively. **(D-G)** Block-average analysis showing the convergence of the error associated with the free energy estimates obtained by metainference metadynamics simulations for the S path variable and the R_g of isolated D1 (panels D-E), and D1-2 (panels F-G). **(H)** Plot of '*angles A-D'* and '*gate distance*' for free energy minima states of D1. **(I)** Plot of '*angles A-D'* and '*gate distance*' for free energy minima states of D1-2. The legend of both panels H and I is indicated on the right of panel I.

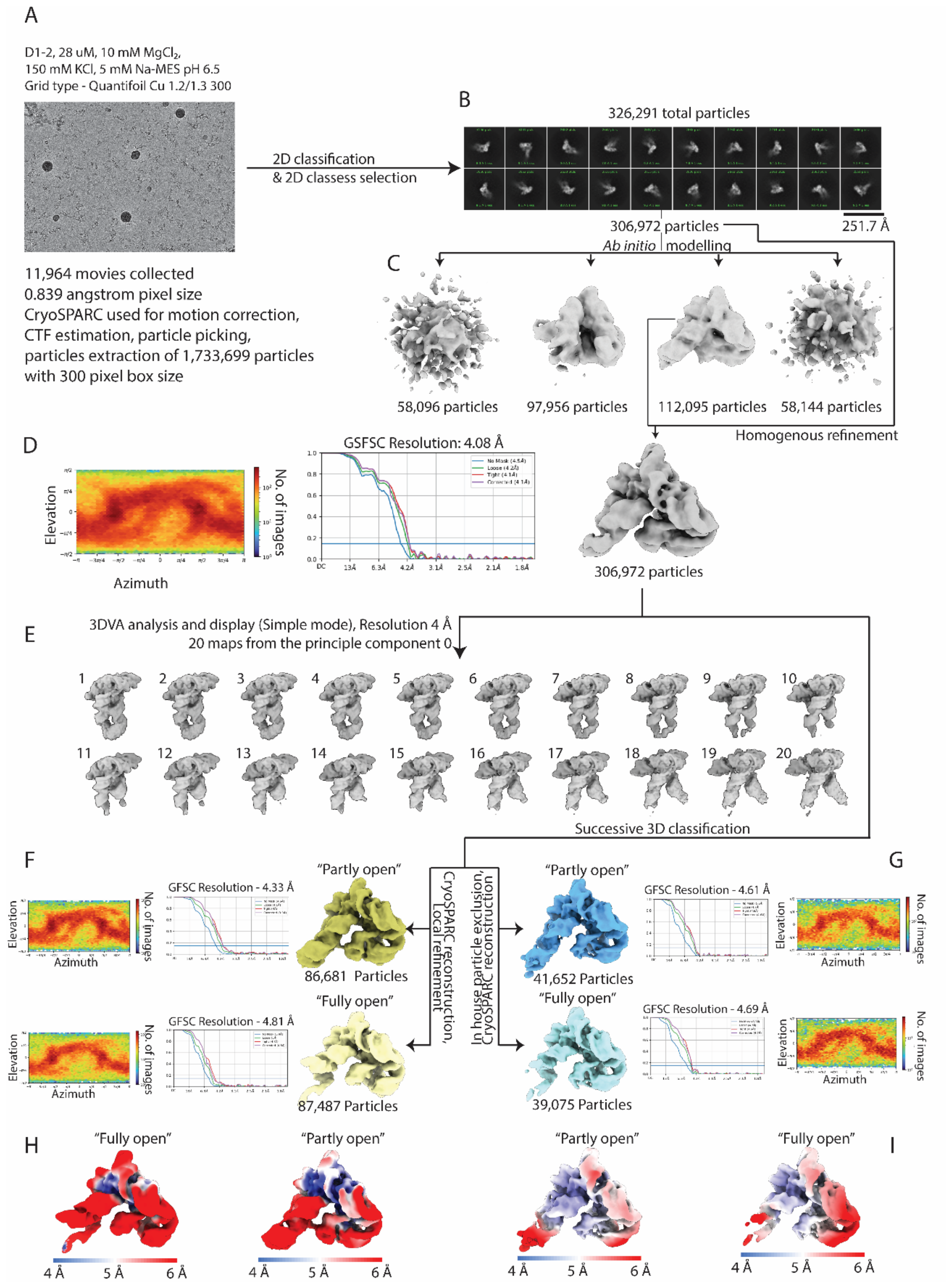

Supplementary Figure 5. D1-2 data processing workflow. (A) Data collection parameters. **(B)** 2D classification. **(C)** *Ab initio* modelling. **(D)** Homogenous reconstruction. **(E)** 3DVA analysis. **(F)** Particles sets representing the "partly open" and "fully open" states, processed by CryoSPARC before manual particle exclusion and sorting. **(G)** Particles sets representing the "partly open" and "fully open" states, processed by CryoSPARC after manual particle exclusion and sorting. Panels F-G also display the Fourier shell correlation (FSC) curve indicating the average resolution of the corresponding map, and the direction distribution plot. **(H-I)** Resulting cryo-EM maps colored according to local resolution.

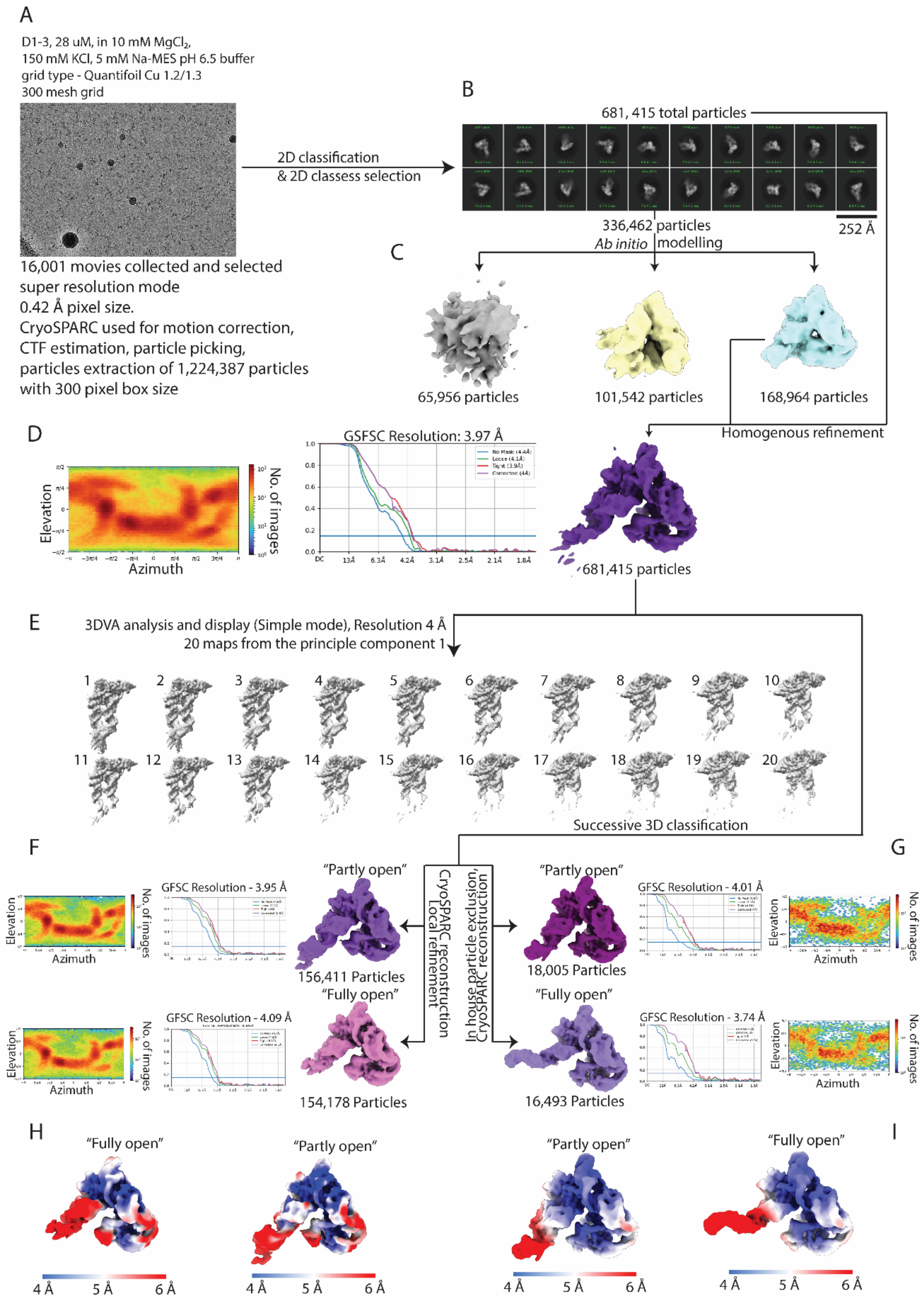

Supplementary Figure 6. D1-3 data processing workflow. **(A)** Data collection parameters. **(B)** 2D classification. **(C)** *Ab initio* modelling. **(D)** Homogenous reconstruction. **(E)** 3DVA analysis. **(F)** Particles sets representing the "partly open" and "fully open" states, processed by CryoSPARC before manual particle exclusion and sorting. **(G)** Particles sets representing the "partly open" and "fully open" states, processed by CryoSPARC after manual particle exclusion and sorting. Panels F-G also display the Fourier shell correlation (FSC) curve indicating the average resolution of the corresponding map, and the direction distribution plot. **(H-I)** Resulting cryo-EM maps colored according to local resolution.

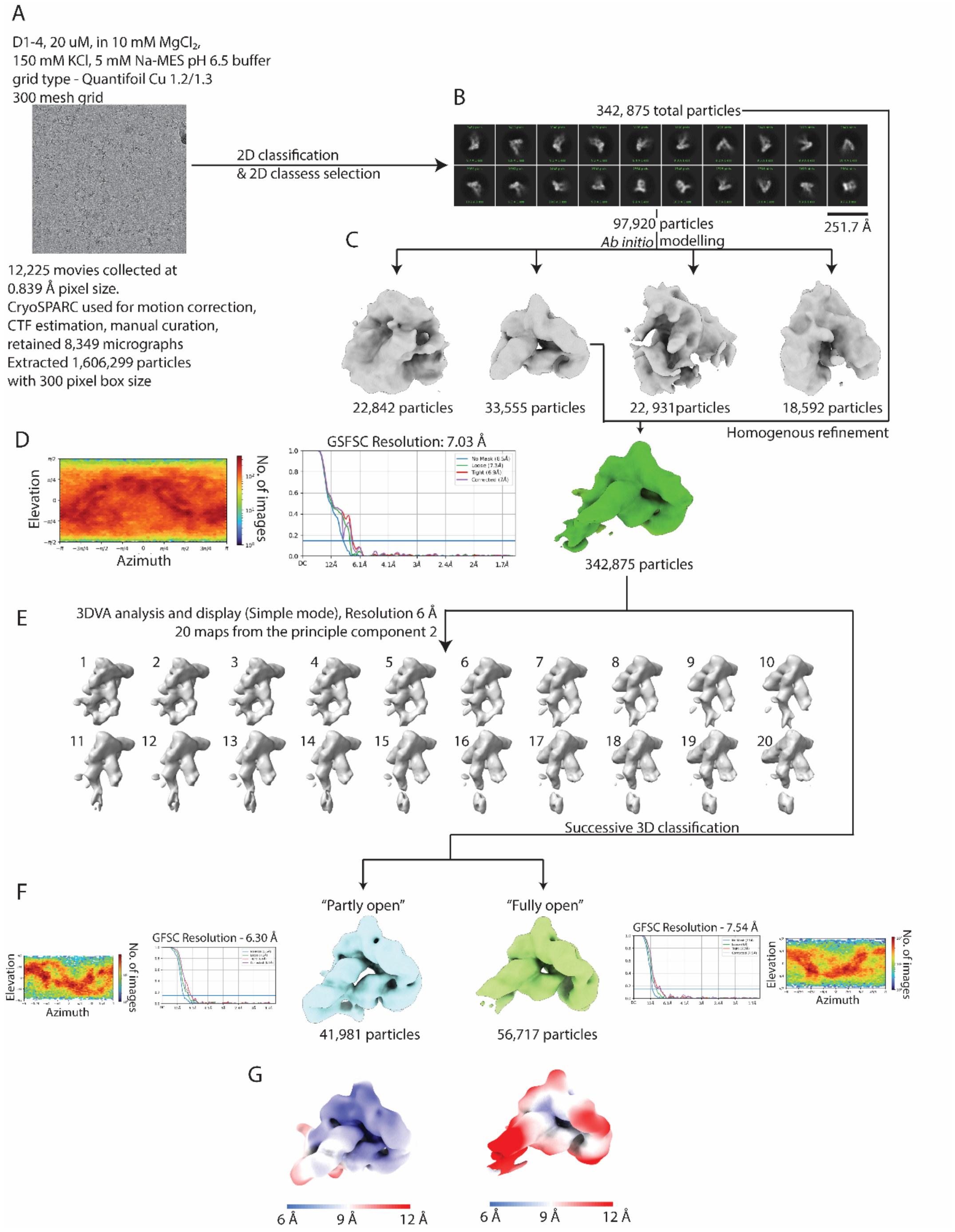

Supplementary Figure 7. D1-4 data processing workflow. (A) Data collection parameters. **(B)** 2D classification. **(C)** *Ab initio* modelling. **(D)** Homogenous reconstruction. **(E)** 3DVA analysis. **(F)** Particles sets representing the "partly open" and "fully open" states, processed by CryoSPARC. For D1-4, due to the lower resolution of the dataset, we did not perform manual particle exclusion and sorting. **(G)** Resulting cryo-EM maps colored according to local resolution.

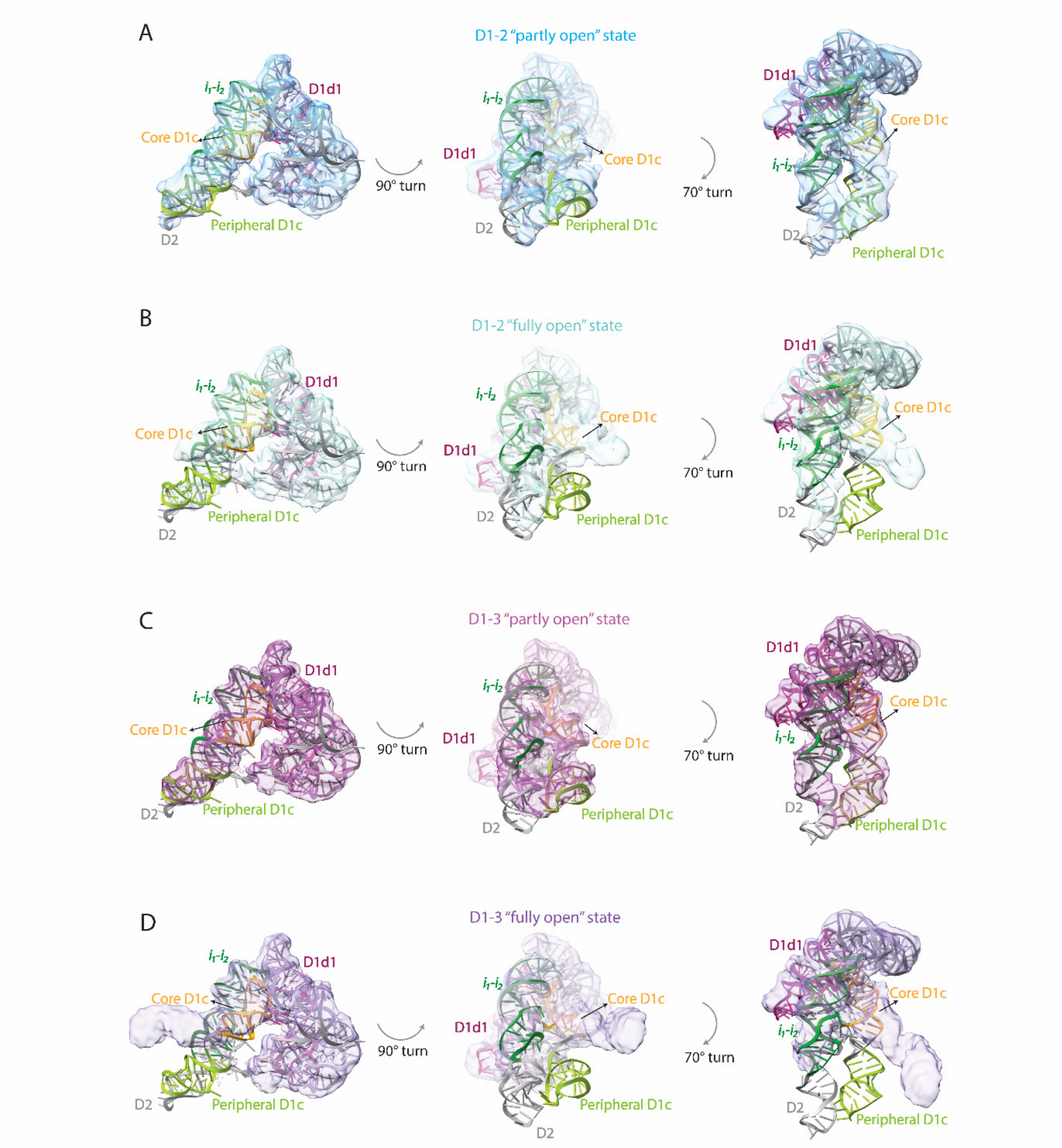

Supplementary Figure 8. Comparison of "partly open", "fully open", and "catalytic" states. (A) Superposition of the density of the "partly open" state of D1-2 and the coordinate model of the "catalytic" state in three different orientations. **(B)** Superposition of density of the "partly open" state of D1-3 and the coordinate model of the "catalytic" state in three different orientations. In both panels, each subdomain is color coded as in **Figure 1**. D3 is not displayed as it is not visible in the density of the "partly open" state of D1-3. **(C)** Superposition of the density of the "fully open" state of D1-2 and the coordinate model of the "catalytic" state in three different orientations. **(D)** Superposition of the density of the "fully open" state of D1-3 and the coordinate model of the "catalytic" state in three different orientations. In both panels, each subdomain is color coded as in **Figure 1**. D3 is not displayed as it is not visible in the density of the "fully open" state of D1-3.

A

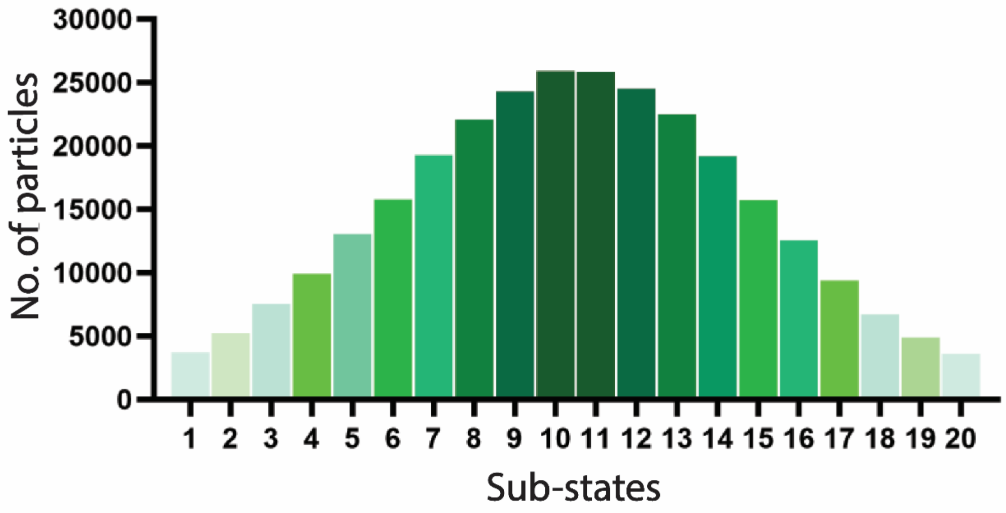

B

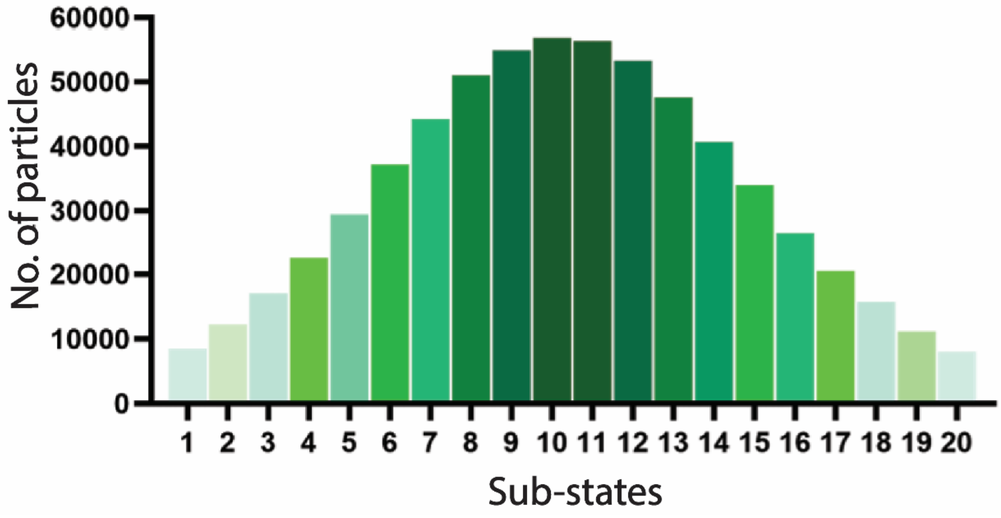

Supplementary Figure 9. Particle distribution of D1-2 and D1-3 across the 3DVA sub-states. **(A)** D1-2 particle distribution across sub-states 1 to 20 obtained from 3DVA. **(B)** D1-3 particle distribution across sub-states 1 to 20 obtained from 3DVA. Source data are provided as a Source Data file.

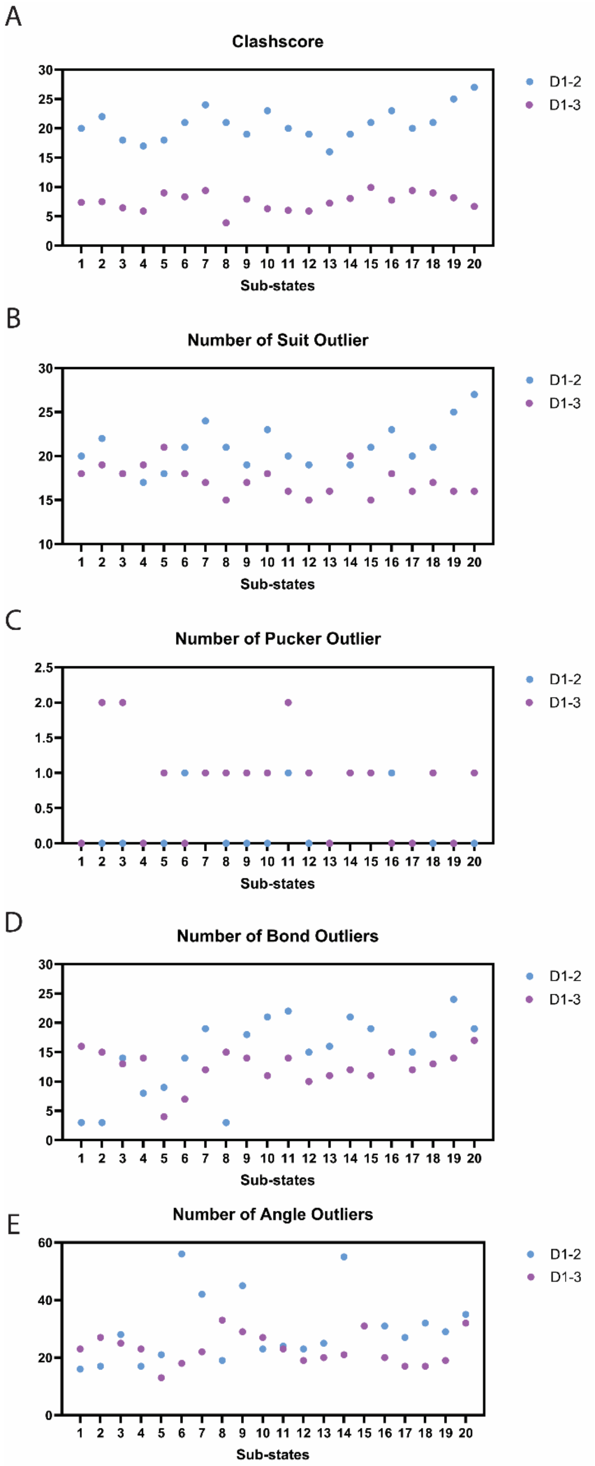

Supplementary Figure 10. Quality of refinement of the 3DVA models. (A) Distribution plot of clash score values of 3DVA sub-state models of D1-2 (blue) and D1-3 (magenta). **(B)** Distribution plot of number of suit outliers of 3DVA sub-state models of D1-2 (blue) and D1-3 (magenta). **(C)** Distribution plot of number of sugar pucker outliers of 3DVA sub-state models of D1-2 (blue) and D1-3 (magenta). **(D)** Distribution plot of number of bond outliers of 3DVA sub-state models of D1-2 (blue) and D1-3 (magenta). **(E)** Distribution plot of number of angle outliers of 3DVA sub-state models of D1-2 (blue) and D1-3 (magenta).

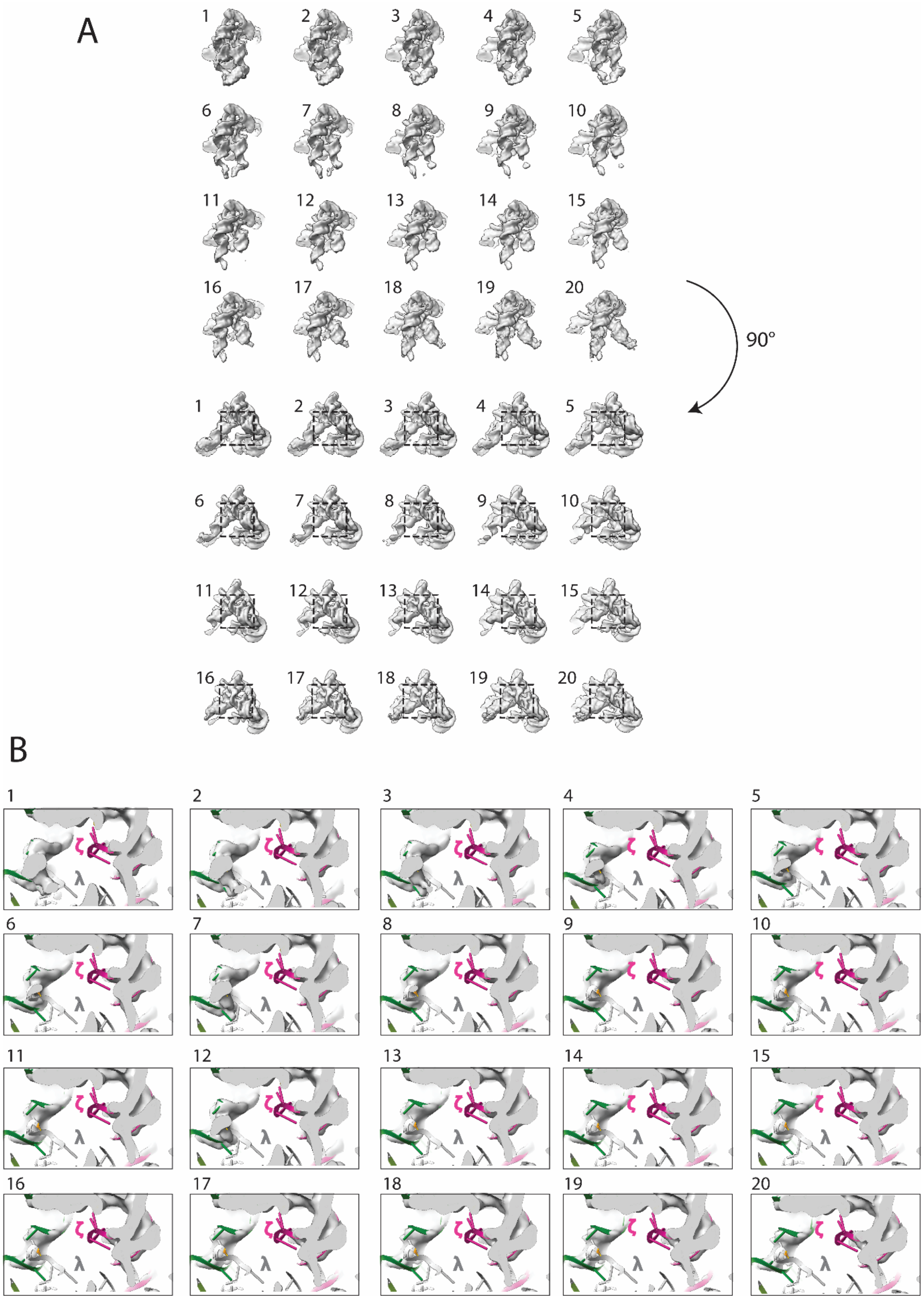

Supplementary Figure 11. Density for λ and ζ motifs in the 3DVA maps of D1-2. (A) 3DVA maps of sub-states 1 to 20 of D1-2. Each map is depicted in two representations, rotated by 90º with respect to each other. The dashed black square identifies the location of the ζ and λ motifs. **(B)** Zoom into the density of the ζ and λ motifs.

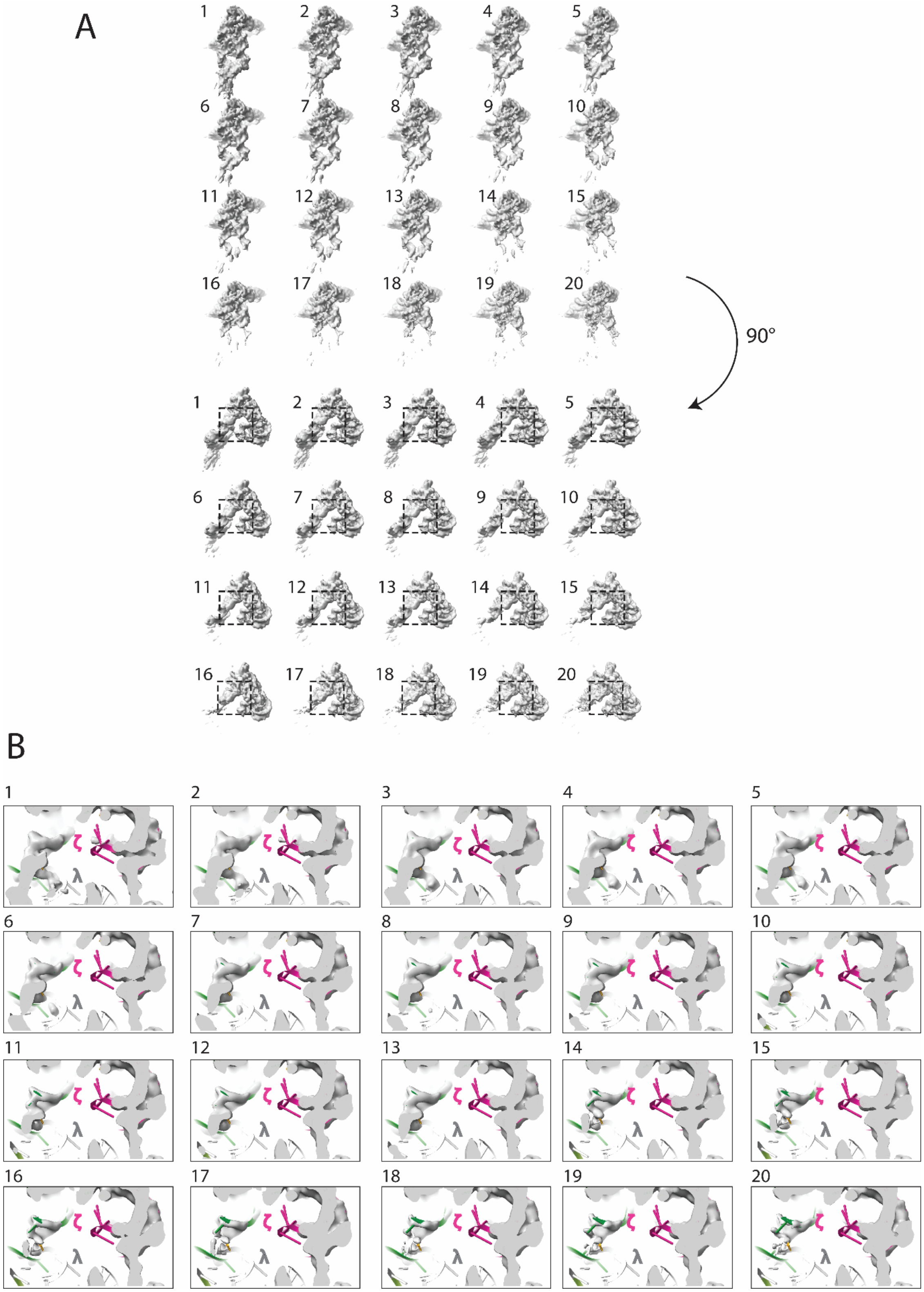

Supplementary Figure 12. Density for λ and ζ motifs in the 3DVA maps of D1-3. (A) 3DVA maps of sub-states 1 to 20 of D1-3. Each map is depicted in two representations, rotated by 90º with respect to each other. The dashed black square identifies the location of the ζ and λ motifs. **(B)** Zoom into the density of the ζ and λ motifs.

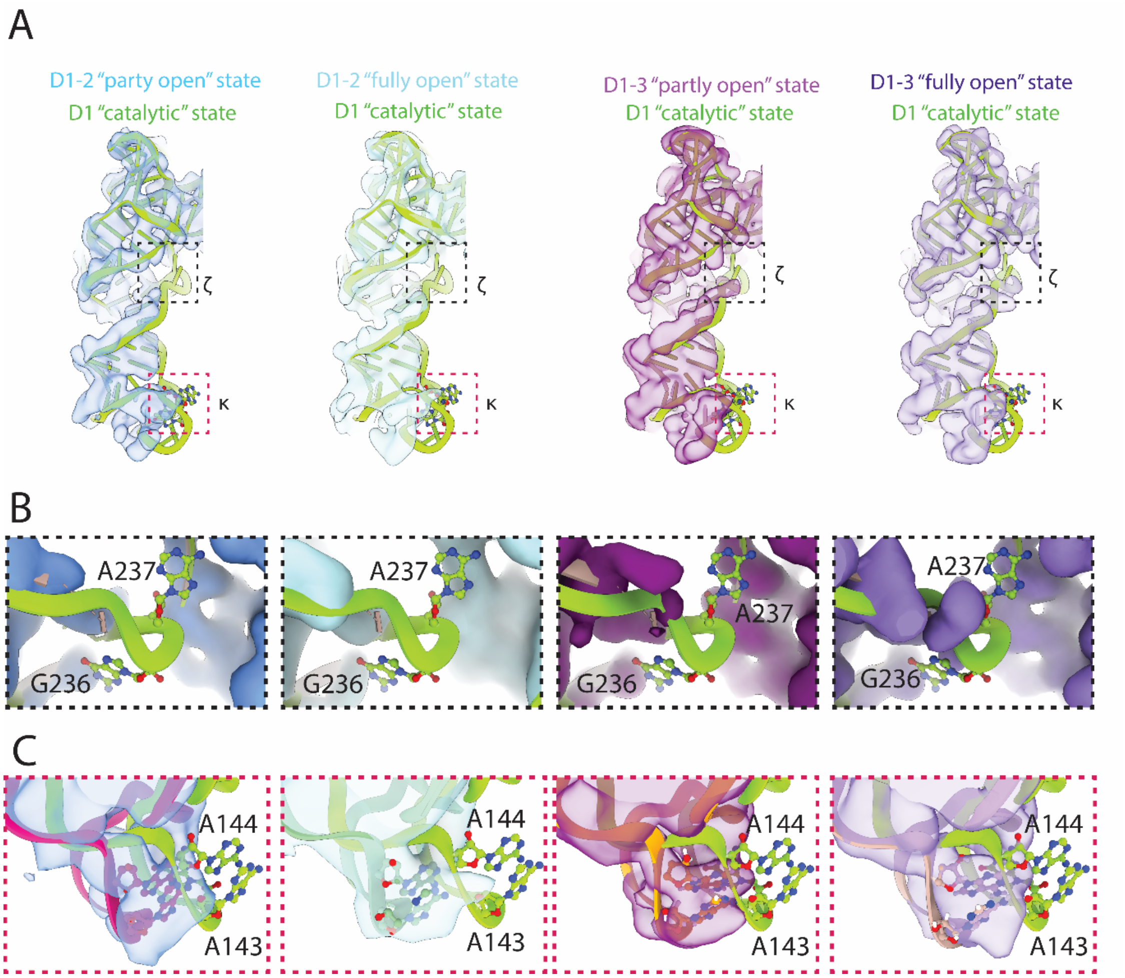

Supplementary Figure 13. Flexibility of the ζ and κ motifs. (A) Superposition of the "catalytic" state (green) on the densities of the D1-2 "partly open" state (blue), of the D1-2 "fully open" state (light teal), of the D1-3 "partly open" state (magenta), and of the D1-3 "fully open" state (purple), respectively, from left to right. The two dashed squares indicate the location of the ζ and κ motifs, respectively. **(B)** Zoom into the superposition of the ζ motif (residues A237 and G236) of the "catalytic" state on the densities of the D1-2 "partly open" state (skyblue), of the D1-2 "fully open" state (light teal), of the D1-3 "partly open" state (magenta), and of the D1-3 "fully open" state (purple), respectively, from left to right. **(C)** Zoom into the superposition of the κ motif (residues A143 and A144) of the "catalytic" state on the densities of the D1-2 "partly open" state (skyblue), of the D1-2 "fully open" state (light teal), of the D1-3 "partly open" state (magenta), and of the D1-3 "fully open" state (purple), respectively, from left to right.

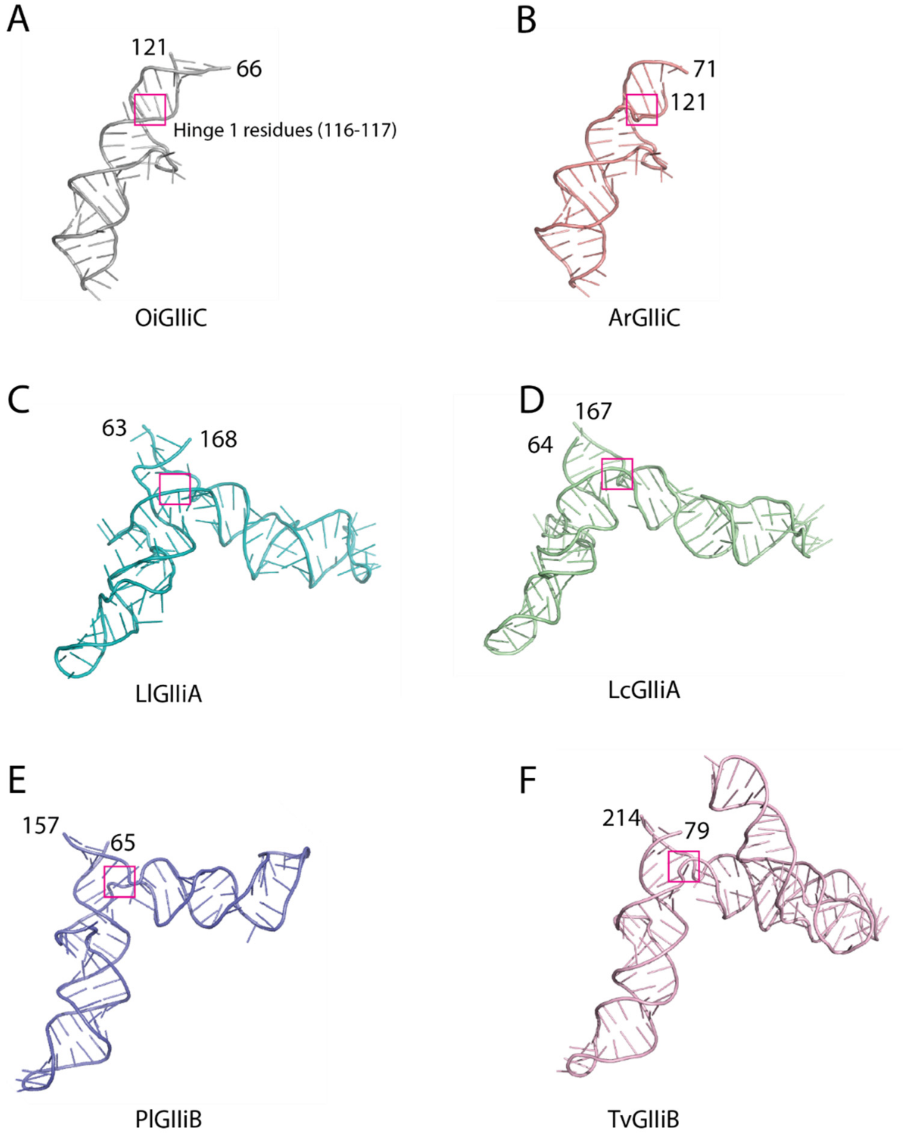

Supplementary Figure 14. Structure of helix D1c across homologous group II introns. (A) Helix D1c in the group IIC intron from *O. iheyensis* (PDB id 4FAQ). **(B)** Helix D1c in the group IIC intron from *Agathobactor rectalis* (PDB id 7UIM). **(C)** Helix D1c in the group IIA intron from *Lactococcus lactis* (PDB id 5G2Y). **(D)** Helix D1c in the group IIA intron from *Lactococcus cremoris* (PDB id 7D0F). **(E)** Helix D1c in the group IIB intron from *Pylaiella littoralis* (PDB id 4R0D). **(F)** Helix D1c in the group IIB intron from *Thermosynechococcus vestitus* (PDB id 8FLI).

SUPPLEMENTARY TABLES

Supplementary Table 1. Conformational descriptors. '*Angles A-D*' and '*gate distance'* are described in **Figure 1** and in the main text. NA = not available, due to the low resolution or disordered features of the corresponding maps.

	**Construct**	**State of D1**	**'*Angle A*' core D1c/ D1d1**	**'*Angle B*' core D1c/ peripheral D1c**	**'*Angle C*' core D1c/ i_1-i_2**	**'*Angle D*' D1d1/ i_1-i_2**	**'*Gate distance*' G75-U238**
**X-ray**	**D1**	Closed	62.9°	51.4°	46.1°	51.7°	32.0 Å
**Cryo-EM**	**D1-2**	Partly open	67.2°	41.5°	36.1°	54.5°	36.8 Å
		Fully open	79.7°	NA	43.5°	57.3°	38.4 Å
	**D1-3**	Partly open	67.2°	40.5°	36.7°	55.0°	36.0 Å
		Fully open	72.3°	NA	39.0°	58.3°	38.5 Å
**X-ray**	**D1-5**	Catalytic	57.0°	40.3°	34.9°	53.6°	34.0 Å

Supplementary Table 2. SAXS data collection. Essential data acquisition, sample details, data analysis, modelling fitting and software for D1, D1-2, D1-3, and D1-4 SAXS datasets.

(*a*) Sample details				
Sample	D1	D1-2	D1-3	D1-4
Organism	*O. iheyensis*	*O. iheyensis*	*O. iheyensis*	*O. iheyensis*
Molecular mass *M* (kDa)	89	96	103	125
Injection volume (µL)	95 µL	95 µL	95 µL	95 µL
Loading conc (mg/mL)	1.13 mg/mL	1.13 mg/mL	1.13 mg/mL	1.13 mg/mL
Flow rate (mL/min)	0.5 mL/min	0.5 mL/min	0.5 mL/min	0.5 mL/min
(*b*) SAXS data collection parameters				
Source, instrument and description or reference – BioSAXS beamline ESRF Grenoble				
Wavelength (Å) - 0.9918				
Beam geometry (size, sample-to-detector distance) - ~ 200 µm x 100 µm				
q-measurement range ($Å^{-1}$ or nm^{-1}) - 0.025 - 6 nm^{-1}				
Absolute scaling method - Comparison with scattering from pure H_2O				
Basis for normalization to constant counts - To transmitted intensity by direct beam counter				
Method for monitoring radiation damage - X-ray dose where relevant				
Exposure time – 2 sec				
Sample temperature (ºC) – 25 °C				
(*c*) Software employed for SAXS data reduction, analysis and interpretation				
SAXS data reduction – Chromix from ATSAS 3.2.1				
Basic analyses: Guinier, *P*(*r*), scattering particle volume - GNOM from ATSAS				
Shape/bead modelling – Dammif (https://www.embl-hamburg.de/biosaxs/dammif.html)				
Molecular graphics **-** PyMoL				
(*d*) Structural parameters				
	D1	D1-2	D1-3	D1-4
Guinier Analysis				
$I_{(0)}$ (cm^{-1})	23.72		21.40	28.05
R_g (Å)	3.84 ± 0.18	3.64 ± 0.05	3.59 ± 0.04	4.15 ± 0.17
q-range ($Å^{-1}$)	0.12-0.23	0.74-1.29	0.18-0.36	0.28-0.34
Quality-of-fit parameter	0.88	0.98	0.98	0.96
M from $I_{(0)}$	113.1	116.8	92.3	157.7
P(*r*) analysis				
$I_{(0)}$ (cm^{-1})	23.71	14.20	21.66	27.57
R_g (Å)	3.76	3.75	3.67	4.12
d_{max} (Å)	12.98	12.04	12.04	12.60
q-range ($Å^{-1}$)	0.11-2.16	3`.76	23-423	11-361
Quality-of-fit parameter	1.08	0.66	0.95	0.62
Volume (V_P, $Å^3$)	187,584	178,575	195,204	246,675
(e) Data and model deposition IDs				
SASBDB code	SADX39	SASDX59	SASDX49	SASDX69

Supplementary Table 3. SAXS data. Hydrodynamics parameters and X^2 value obtained for D1, D1-2, D1-3 and D1-4 by SEC-SAXS and SAXS- and EM-driven MD simulations. Model structures of D1-2, D1-3 and D1-4 for *in silico* estimation were obtained from the D1-5 crystal structure (PDB id 4FAQ).

Construct	Parameter	*In silico* estimated value	SEC-SAXS experimental value
**D1**	R_g (nm)	3.38	3.84 ± 0.18
	D_{max} (nm)	9.46	12.98
	X^2_{crysol} / X^2_{MD}		1.77 / 1.62
**D1-2**	R_g (nm)	3.52	3.64 ± 0.05
	D_{max} (nm)	11.96	11
	X^2_{crysol} / X^2_{MD} / X^2_{3DVA}		1.55 / 1.70 / 1.26
**D1-3**	R_g (nm)	3.70	3.59 ± 0.04
	D_{max} (nm)	12.22	12.04
	X^2_{crysol} / X^2_{3DVA}		1.14 / 1.04
**D1-4**	R_g (nm)	3.88	4.15 ± 0.17
	D_{max} (nm)	12.41	12.60
	X^2_{crysol}		1.38

Supplementary Table 4. Kinetics rate constants. Splicing rate constants of the first (k_1) and second (k_2) steps of splicing of *O. iheyensis* I1 group II intron (pOiA and related pOiA-hinge1mut) and v_{max} and K_M values of *S. cerevisiae* ai5γ group II intron (ai5γ-D135 and ai5γ-D135-hinge1mut). Errors represent standard errors of the mean (s.e.m.) calculated from n = 3 independent experiments.

O. iheyensis	**pOiA**	**pOiA-hinge1mut**
**k_1 (min^{-1})**	0.016 ± 0.003	0.003 ± 0.000
**k_2 (min^{-1})**	0.041 ± 0.007	0.028 ± 0.005
S. cerevisiae	**ai5γ-D135**	**ai5γ-D135-hinge1mut**
**v_{max} ($nmol \cdot L^{-1} \cdot min^{-1}$)**	22.47 ± 9.49	0.74 ± 0.06
**K_M ($nmol \cdot L^{-1}$)**	192.67 ± 80.93	7.87 ± 3.24

Supplementary Table 5. Names and sequences of primers used in the study. Primers 1 and 2 were used to clone D1-2, primers 1 and 3 were used to clone D1-3, primers 4 and 5 were used to clone D1-4, primers 6 and 7 were used to clone pOiA-hinge1mut, and primers 8 and 9 were used to clone ai5γ-D135-hinge1mut.

No.	Primer name	Sequence
**1**	SLIC_D1-2J_primer_1'	5’-GGC GAT TAA GTT GGG TAA CGC CAG GGT TTT CCC AGT CAC G
**2**	SLIC_D1-2J_primer_2'	5’-GCA GGT CGA CTC TAG AGG ATC CGC TGA CTT CTG TTT CCA G
**3**	SLIC_D1-2J3_primer_2'	5’-GCA GGT CGA CTC TAG AGG ATC CTT CCG TCC TTC CCC TGC G
**4**	pSJ05_D4_addition_fw_pri	5’-CGC GCC TAA GCT TGA GGA TCC TCT AGA GTC GAC C
**5**	pSJ05_D4_addition_rv_pri	5’-GGT CGA CTC TAG AGG ATC CTC AAG CTT AGG CGC G
**6**	pOiA_Hinge_1_F	5’-GGC AGA AGT AAC AGT TAG CCG TCC GCA AGG GTG TCC G
**7**	pOiA_Hinge_1_R	5’-CGG ACA CCC TTG CGG ACG GCT AAC TGT TAC TTC TGC C
**8**	D135_hinge_F	5’-GGT AAC ATA AAT ATG CTA AGC TGT AAT TAA AAG TAT CC
**9**	D135_hinge_R	5’-GGA TAC TTT TAA TTA CAG CTT AGC ATA TTT ATG TTA CC

Supplementary Table 6. MD simulations checklist.

**Reliability and reproducibility checklist for molecular dynamics simulations**<br>***All boxes must be marked YES by acceptance unless an N/A option is available**	**Yes**	**N/A**	**Response (Please state where this information can be found in the text)**
**1. Convergence of simulations and analysis**			
1a. Is an evaluation presented in the text to show that the property being measured has equilibrated in the simulations (*e.g.* time-course analysis)?	☒		Methods "MD simulations set up"
1b. Then, is it described in the text how simulations are split into equilibration and production runs and how much data were analyzed from production runs?	☒		Methods "MD simulations set up"
1c. Are there at least 3 simulations per simulation condition with statistical analysis?	☒		Methods "Metainference metadynamics"
1d. Is evidence provided in the text that the simulation results presented are independent of initial configuration?	☒		Methods "Metainference metadynamics"
**2. Connection to experiments**			
2a. Are calculations provided that can connect to experiments (*e.g.* loss or gain in function from mutagenesis, binding assays, NMR chemical shifts, J-couplings, SAXS curves, interaction distances or FRET distances, structure factors, diffusion coefficients, bulk modulus and other mechanical properties, *etc.*)?	☒		Results "Conformational dynamics of group II intron domain 1 (D1) in solution"
**3. Method choice**			
3a. Is it described in the text what force field and water model are used and why?	☒		Methods "MD simulations set up"
3b. Do simulations contain membranes, membrane proteins, intrinsically disordered proteins, glycans, nucleic acids, polymers, or cryptic ligand binding?	☐	☒	Response not needed if **N/A**
If 3b is **YES**, are enhanced sampling methods used?	☐	☒	Response not needed if **N/A**
If enhanced sampling methods are used, are the convergence criteria clearly stated?	☒		Methods "MD simulations set up"
If 3b is **YES**, is it explained in the text why or why not enhanced sampling methods are used?	☒		Results "Conformational dynamics of group II intron D1 in solution"
**4. Code and reproducibility**			
4a. Is a table provided describing the system setup, such as simulation box dimensions, total number of atoms, total number of water molecules, salt concentration, lipid composition (number of molecules and type)?	☒		
4b. Is it described in the text what simulation and analysis software and which versions are used?	☒		Methods "MD simulations set up"
4c. Are initial coordinate and simulation input files and a coordinate file of the final output provided as supplementary files or in a public repository?	☒		
4d. Is there custom code or custom force field parameters?	☐	☒	Response not needed if **N/A**
If **YES**, are they provided as supplementary profiles or in a public repository?	☐		
