# Supplementary material for: Dynamic assembly of a large multidomain ribozyme visualized by cryo-electron microscopy
Source: Nat Commun. 2025 Nov 27;16:10195. doi: 10.1038/s41467-025-65502-8 (PMC12660903; doi:10.1038/s41467-025-65502-8)
Supplement: Supplementary file 2 — Description of Additional Supplementary Files [file 41467_2025_65502_MOESM2_ESM.pdf]

Description of Additional Supplementary Files

File Name: Supplementary Data 1
File Description: Molecular Dynamics simulation data

File Name: Supplementary Movie 1
File Description: Group II intron assembly mechanism. The movie reports the assembly mechanism of O. iheyensis group II intron, from its pre-folded “closed” scaffold D1 to its “catalytic” state D1-5, through “open” states formed by D1-2, D1-3, and D1-4, as captured by the cryo-EM structures described in this work.
